# The impact of sociodemographic and health aspects on cognitive performance in the older adult population in the republic of Serbia

**DOI:** 10.3389/fpubh.2024.1384056

**Published:** 2024-07-10

**Authors:** Andrea Mirkovic, Svetlana Radevic, Snezana Radovanovic, Ivana Simic Vukomanovic, Katarina Janicijevic, Sanja Ilic, Ognjen Djordjevic, Gordana Djordjevic, Jovana Radovanovic, Viktor Selakovic, Nikola Savic, Andjela Gogic

**Affiliations:** ^1^Faculty of Medical Sciences, University of Kragujevac, Kragujevac, Serbia; ^2^Faculty of Medical Sciences, Department of Social Medicine, University of Kragujevac, Kragujevac, Serbia; ^3^Faculty of Medical Sciences. Department of Epidemiology, University of Kragujevac, Kragujevac, Serbia; ^4^Faculty of Health and Business Studies, Department of Health Studies, Singidunum University, Valjevo, Serbia; ^5^Faculty of Medical Sciences, Department of Medical Statistic, University of Kragujevac, Kragujevac, Serbia

**Keywords:** cognitive abilities, sociodemographic factors, health outcomes, older adult, cognitive functions, health survey

## Abstract

The aim of this study was to investigate how sociodemographic and health factors contribute cognitive abilities in the older population of the Republic of Serbia, using data from the 2019 national health survey. The study included 3,743 participants, of whom 2,061 (55.1%) were women and 1,682 (44.9%) were men. The median age of all participants was 72 (10) years. Study used logistic regression on cross-sectional data to analyze how education, social support, and healthcare access affect cognitive abilities, while adjusting for demographic variables. The results revealed negative associations between higher levels of education and lower odds of experiencing memory and concentration problems, while recent visits to specialists were positively associated with increased risk for the same. The highest percentage of participants (22.6%) reporting major difficulties in memory and concentration were in the age group of 85–89 years (*p* < 0.001). A statistically significant relationship was found between social support and issues related to memory and concentration (*p* < 0.001). Social support emerged as a significant factor in preserving cognitive abilities. The discussion underscores the need for a comprehensive approach in promoting cognitive health, taking into account education, social integration, and access to healthcare as key factors. The study acknowledges its limitations, including its cross-sectional nature and potential subjective biases in self-assessment of cognitive abilities. Future research should incorporate longitudinal studies and more objective measures of cognitive abilities.

## 1 Introduction

In the past few decades, the number of older adults worldwide has dramatically increased, and according to the World Health Organization (WHO) forecasts, there is expected to be a threefold increase in the number of people aged 80 and over by 2050, reaching around 426 million globally (1). Like many other countries, Serbia is facing demographic changes that require adjustments to policies and strategies to meet the needs of the older population. The average age of the population in Serbia is 43.2 years (2), significantly higher compared to the global average of 30.3 years according to research by the World Economy (3). The aging process leads to physical and mental decline, affecting emotional and cognitive functioning, which can result in reduced social activity and exposure to numerous health risks (4). WHO defines health as a state of complete physical, mental, and social well-being, not merely the absence of disease or infirmity (5). As key elements of mental health, WHO lists achieving personal potential, effective coping with everyday stressors, and contributing to the community (6). Cognitive impairment is defined as a decline in memory, learning new information, and concentration, which affects daily activities and functioning (7). Previous studies have indicated the contribution of various factors on cognitive ability throughout life (4, 8–10). Cognitive impairment includes a decline in memory, learning, and concentration, affecting the daily activities of individuals facing it, especially in old age (4, 7–10). Various factors, such as sociodemographic characteristics, have shown a significant impact on the cognitive abilities of older adults, regardless of the specific cognitive tests applied in studies (9). Lower literacy is associated with poorer cognitive performance, while socio-economic status can contribute to better cognitive health (7–11). Higher socioeconomic status, including factors such as income and occupation, is associated with better access to healthcare, healthier lifestyles, and reduced stress, all of which can contribute to better cognitive health in old age (9). Previous studies suggest that social participation is a protective factor for mental stimulation (11–14), while social isolation and the presence of chronic diseases can contribute to depression and reduced cognitive abilities (13, 14). A close relationship has been observed between age and the onset of dementia, which is generally associated with accelerated cognitive decline as age progresses (12). Pain is an underexplored cause of cognitive decline in older adults (15–18). Cognitive decline and dementia can make experiencing pain and expressing it more difficult, presenting a challenge for healthcare workers and caregivers of older adults (15).

This study aims to comprehensively explore the complex relationships between different factors such as age, gender, education, socioeconomic status, social engagement, presence of chronic diseases, and pain perception in the context of cognitive functions in older adults in Serbia. Through this analysis, we hope to lay the groundwork for a better understanding of these interactions not only within our national context, which could contribute to the development of better strategies and policies to promote cognitive health in the older population.

## 2 Materials and methods

### 2.1 Study design

The research was designed as a national population-based two-stage cross-sectional study and was conducted as part of the fourth national survey, the “Health Survey of the Population of Serbia in 2019.” This comprehensive study was carried out by the Statistical Office of the Republic of Serbia in collaboration with the Institute of Public Health of Serbia “Dr Milan Jovanović Batut” and the Ministry of Health of the Republic of Serbia. The Ministry of Health obtained approval for the use of the questionnaire from the European Commission. All participants were informed about the research’s goal and gave their consent. Ethical standards in health research were aligned with international standards (Declaration of Helsinki by the World Medical Association) and the country’s specific legislation. The study’s methodology adhered to the European Health Interview Survey (EHIS Wave 3) (19) standards, ensuring robust and internationally recognized research practices. The ethical considerations were paramount, with researchers obligated to provide participants with a printed document detailing the research, ethical committee approval, respondents’ rights, and information on how and where to file complaints/grievances if they believed their rights were compromised. Furthermore, the University of Kragujevac was granted access to the National Health Survey 2019 database for scientific research purposes, as officially communicated through a letter from the Institute of Public Health of the Republic of Serbia “Dr. Milan Jovanović Batut.” This collaboration reflects a meticulous and transparent approach, aligning with the highest standards in research ethics and methodology.

### 2.2 Selection criteria

The analysis is based on a sample of 3,705 participants aged 65 and above. The research spanned 3 months, from October to December, in 2019. The sample encompasses all households listed in all enumeration areas during the 2011 Census. To achieve a random sample of households and respondents, a combination of two sampling techniques was employed: stratification and multistage sampling. Stratification was carried out based on the type of settlement (urban and other) and four regions: Belgrade Region, Vojvodina Region, Šumadija and Western Serbia Region, and Southern and Eastern Serbia Region. A sample of 5,114 households was realized, totaling 15,621 respondents. It is noteworthy that individuals placed in social institutions and prison facilities were excluded from the research.

### 2.3 Measurement instruments

Participant data, including demographic and socioeconomic information, were collected through personal interviews conducted at home. Household information was obtained through a household questionnaire, part of validated instruments based on standard surveys. Independent variables included demographic characteristics (age, gender, type of settlement, marital status) and socioeconomic status (education and household wealth index). Participant age was categorized into age groups (65–69, 70–74, 75–79, 80–84, 85–89, 90+), while gender, were defined as male or female, place of residence, urban or rural, and marital status, married, and unmarried/single, cohabiting, divorced, or widowed, respectively. Variables reflecting socioeconomic status included education (no schooling, incomplete primary school, secondary school, higher education, master’s or doctorate) and household wealth index.

#### 2.3.1 Cognitive functional limitation

Cognitive functional impairments were analyzed to assess participants’ ability in relation to daily functioning concerning the degree of memory and concentration impairment. The related question asked was: “Do you have difficulty with memory or concentration?” The provided response options were: No difficulty, with minor difficulties, with major difficulties, unable.

#### 2.3.2 Social support

The social support score was formed by assigning and summing points for each response to three possible questions from the “Oslo-3 Social Support Scale” (20): “How many people are so close to you that you can count on them when you have serious personal problems?” [points range from 1 (“None”) to 4 (“6 or more”)], “How much are people really interested in you, in what you do, what happens in your life?” [points range from 1 (“Not at all interested”) to 5 (“Very interested”)], “How easy is it to get practical help from neighbors/friends if you need it?” [points range from 1 (“Very difficult”) to 5 (“Very easy”)]. By summing points on these three questions, a social support score was established: strong social support (12–14 points), moderate (9–11 points), and poor (3–8 points).

#### 2.3.3 Mental health

To assess the presence of depression, the PHQ-8 questionnaire (Patient Health Questionnaire-8) was used as a diagnostic tool. It consists of eight items related to specific mental problems and distress. A PHQ-8 score ranging from zero to four indicates no symptoms of depression, a score from five to nine indicates mild symptoms of depression, and a total PHQ-8 score of ten and above indicates a high probability of depression, further classified as moderate (PHQ-8 score from 10 to 14), moderately severe (PHQ-8 score from 15 to 19), and severe depressive episode (PHQ-8 score 20 and above).

#### 2.3.4 Wealth index

The Demographic and Health Survey Wealth Index, or wealth index estimation, has been extensively described in previous studies and includes variables related to property, excluding income (21). Well-being index - a complex measure of household cumulative living standards, calculated using data on household ownership of selected assets such as televisions and bicycles; materials used for housing construction; and types of access to water and sanitation facilities. Household wealth in Serbia is ranked into five socio-economic categories (5- wealthiest, 4- rich, 3- middle class, 2- poor, and 1- poorest).

### 2.4 Self-assessment of health - minimum European health module

Subjective measurement, or self-assessment of health, contributes significantly to the overall evaluation of health issues, disease burden, and healthcare needs at the national level. This assessment does not replace objective indicators, derived from routine health statistics, but rather complements them. Research has shown that self-assessment of health is a significant predictor of mortality in the population, with the basic question about health defined by the WHO. In addition, regarding the impact of pain, respondents were asked two questions: “Have you had any pain in the past four weeks?”; “During the past four weeks, how much has pain interfered with your normal work (including both work outside the home and housework)?” Participants were asked to self-assess their health using the question: “A. How would you rate your overall health?” These indicators, including the self-assessment of health and pain interference, are crucial for shaping EU health policies and monitoring the health status of the population. Certain variables measured in this research are also intended to be used as standardized key variables in other social surveys conducted both in the EU and in Serbia (22).

### 2.5 Statistical analysis

Data processing utilized SPSS 23.0 on Windows. Descriptive and inferential statistics, including *χ*^2^ tests and logistic regression, were applied for categorical data analysis. The *χ*^2^ test assessed single characteristic distribution, while rxk-type contingency tables examined differences in two or more features. Bivariate and multivariate logistic regression explored the relationship between dependent and significant independent variables (*p* ≤ 0.05). In constructing the multivariate regression model, we first performed univariate analyses to identify variables significantly associated with the outcome. We chose to not include all statistically significant from each domain variables in the multivariate model; instead, a single representative variable from each domain (excluding sociodemographic characteristics) was chosen. This technique was premised on the fact that variables within a domain tended to capture similar constructs and including all of them could result in multicollinearity. We thereby sought to develop a more interpretable model, as well as one that accounted for the main features of each domain in appropriate detail through selection of single variables from among many candidate explanatory factors. Results, presented in tables considered significance at a 5% probability level to assess the contribution of potential confounding variables on the associations between the independent variable and the outcome variable, a confounding analysis was conducted. Specifically, changes of approximately 10% or more in the magnitude of the effect estimates were deemed indicative of a potentially important confounding effect.

## 3 Results

The study included a total of 3,743 participants, of whom 2,061 (55.1%) were women and 1,682 (44.9%) were men. The median age of all participants was 72 (10) years, with the youngest participant being 65 years old and the oldest being 99 years old. Women were older than men, with a median age of 72 (11) years compared to men’s median age of 71 (10) years (*p* < 0.01). Regarding memory and concentration, one in four participants reported minor difficulties, one in twenty reported major difficulties, while only one in a hundred responded “Unable.” The sociodemographic characteristics are presented in Table 1.

**Table 1:** Distribution of sociodemographic characteristics by memory and concentration difficulties in the older adult.

	Do you have difficulty with memory or concentration?					
Variables	No difficulty	With minor difficulties	With major difficulties	Unable	Total (*N*)	p*
**Gender**						
Male	47.8%	39.1%	39.8%	22.5%		<0.001
Female	52.2%	60.9%	60.2%	77.5%		
**Total (N)**	2,577	937	186	40	3,740	
**Missing values**	3					
**Age group**						
65–69	43.4%	22.6%	13.4%	5.0%		<0.001
70–74	27.4%	23.8%	14.0%	12.5%		
75–79	15.6%	20.4%	20.4%	27.5%		
80–84	9.7%	20.1%	22.0%	30.0%		
85–89	3.0%	10.4%	22.6%	17.5%		
90+	0.9%	2.8%	7.5%	7.5%		
**Total (*N*)**	2,577	937	186	40	3,740	
**Missing values**	3					
**Marital status**						
Single	2.3%	1.6%	2.2%	2.5%		<0.001
Married/common law	62.9%	48.9%	45.7%	22.5%		
Widiwed	30.8%	46.4%	51.6%	72.5%		
Divorced	3.9%	3.1%	0.5%	2.5%		
**Total (N)**	2,574	937	184	40	3,735	
**Missing values**	8					
**Place of residence**						
City	54.9%	51.5%	53.2%	70.0%		0.066
Another pleace	45.1%	48.5%	46.8%	30.0%		
**Total (*N*)**	2,577	937	186	40	3,740	
**Missing values**	3					
**Education level**						
No formal education	2.7%	10.4%	22.7%	25.0%		<0.001
Incomplete primary school	12.0%	20.9%	27.6%	27.5%		
Primary school	24.1%	28.8%	24.3%	32.5%		
Secondary school	43.8%	29.8%	21.1%	15.0%		
Higher or vocational school	15.8%	9.8%	4.3%	0.0%		
Master’s or doctorate	1.6%	0.2%	0.0%	0.0%		
**Total (N)**	2,577	936	185	40	3,738	
**Missing values**	5					
**Wealth index**						
1-Poorest	17.6%	25.1%	27.4%	17.5%		<0.001
2- Poor	20.0%	25.9%	24.2%	30.0%		
3- Middle class	22.7%	20.4%	23.1%	22.5%		
4- Rich	21.5%	15.9%	16.7%	17.5%		
5- Wealthiest	18.2%	12.7%	8.6%	12.5%		
**Total (*N*)**	2,577	937	186	40	3,740	
**Missing values**	3					

The highest percentage of participants reporting major difficulties in memory and concentration, nearly one in five, were in the age group of 85–89 years (*p* < 0.001). One in three respondents who indicated experiencing significant challenges with memory and concentration also displayed symptoms of depression. Individuals with severe depressive symptoms showed the highest rate of major difficulties with memory or concentration, nearly two in five (*p* < 0.001). There is a statistically significant relationship between social support and issues related to memory and concentration (*χ*^2^ = 449.871, df = 15, *p* < 0.001). Additionally, participants who underwent hospitalization or sought specialist care within the past year reported a heightened prevalence of memory or concentration difficulties (*p* < 0.001, respectively) (Table 2).

**Table 2:** Distiribution of selected health aspects by memory and concentration difficulties in the older adult.

	Do you have difficulty with memory or concentration?					
Variables	No difficulty	With minor difficulties	With major difficulties	Unable	Total (*N*)	*p* ^*^
**Mental health**						
No symptoms of depression	91.90%	62.60%	25.20%	15.40%		<0.001
Mild symptoms of depression	7.10%	27.60%	38.60%	15.40%		
Moderate depressive episode	0.50%	7.20%	20.50%	15.40%		
Moderately severe depressive episode	0.40%	1.70%	12.60%	38.50%		
Severe depressive episode	0.10%	0.90%	3.10%	15.40%		
**Total (*N*)**	2,523	884	127	13	3,547	
**Missing values**	196					
**Social support**						
Poor social support	87.30%	83.30%	70.40%	76.90%		<0.001
Moderate social support	12.50%	16.50%	28.00%	23.10%		
Strong social support	0.30%	0.20%	1.60%	0.00%		
**Total (*N*)**	2,480	860	125	13	3,478	
**Missing values**	265					
**How is your overall health?**						
Very good	4.20%	0.70%	0.00%	0.00%		<0.001
Good	36.10%	14.90%	7.50%	0.00%		
Fair (neither good nor bad)	41.00%	45.00%	21.10%	0.00%		
Poor	15.90%	31.20%	49.60%	30.80%		
Very poor	2.80%	8.20%	21.80%	69.20%		
**Total (*N*)**	2,527	888	133	13	3,561	
**Missing values**	182					
**Do you have any long-term illness or long-term health problem (…lasting or expected to last at least 6 months)?**						
Yes	73.10%	84.60%	89.80%	95.00%		<0.001
No	26.90%	15.40%	10.20%	5.00%		
**Total (N)**	2,576	935	186	40	3,737	
**Missing values**	6					
**Due to a health problem. Are you limited in performing activities that other people usually do?**						
Severely limited	12.50%	28.20%	70.40%	100.00%		<0.001
Limited. but not severely	33.30%	45.60%	22.00%	0.00%		
Not limited at all	54.30%	26.30%	7.50%	0.00%		
**Total (N)**	2,573	937	186	40	3,736	
**Missing values**	7					
**Have you been limited for at least the past six months?**						
Yes	76.50%	87.20%	94.80%	100.00%		<0.001
No	23.50%	12.80%	5.20%	0.00%		
**Total (N)**	1,175	690	172	40	2077	
**Missing values**	1,666					
**In the past 12 months. Have you been hospitalized (stayed in the hospital overnight or longer)?**						
Yes	11.10%	18.60%	25.30%	15.00%		<0.001
No	88.90%	81.40%	74.70%	85.00%		
**Total (N)**	2,576	936	186	40	3,738	
**Missing values**	5					
**Thinking about all these occasions when you were admitted to the hospital. How many total nights did you spend in the hospital?**						
Up to one month	90.70%	90.3%	93.0%	80.0%		0.806
Over one month	9.30%	9.70%	7.00%	20.0%		
**Total (N)**	279	165	43	5	492	
**Missing values**	3,251					
**In the past 12 months. Have you been admitted to the hospital as a day patient?**						
Yes	8.90%	9.30%	22.80%	12.50%		<0.001
No	91.10%	90.40%	77.20%	87.50%		
**Total (N)**	2,576	933	184	40	3,733	
**Missing values**	10					
**When did you personally last visit/consult a general practitioner**						
Less than 12 months ago	81.60%	84.80%	81.60%	65.00%		0.008
More than 12 mounts ago	17.90%	15.10%	17.30%	35.00%		
Never	0.50%	0.10%	1.10%	0.00%		
**Total (N)**	2,556	928	185	40	3,709	
**Missing values**	34					
**When did you personally last visit a specialist?**						
Less than 12 months ago	53.00%	60.50%	63.00%	55.00%		<0.001
More than 12 mounts ago	41.30%	37.00%	32.00%	45.00%		
Never	5.70%	2.50%	5.00%	0.00%		
**Total (N)**	2,544	908	181	40	3,673	
**Missing values**	70					
**Have you experienced any pain in the past four weeks?**						
None	52.80%	27.80%	18.00%	23.10%		<0.001
Very mild	15.50%	15.10%	11.30%	0.00%		
Mild	12.30%	19.40%	12.80%	7.70%		
Moderate	13.10%	24.00%	30.80%	23.10%		
Severe	4.80%	10.80%	22.60%	30.80%		
Very severe	1.60%	2.90%	4.50%	15.40%		
**Total (N)**	2,528	888	133	13	3,562	
**Missing values**	181					
**In the past four weeks. How much has pain interfered with your normal work (including both work outside the home and housework)?**						
Not a lot	12.90%	8.00%	3.70%	0.00%		<0.001
A litte	41.30%	26.70%	17.60%	0.00%		
Moderately	24.60%	34.90%	30.60%	0.00%		
Quite a bit	14.90%	20.60%	24.10%	40.00%		
Very much	6.30%	9.80%	24.10%	60.00%		
**Total (N)**	1,193	641	108	10	1952	
**Missing values**		1791				

Analyzing the statistically significant predictors of functional limitations regarding memory or concentration, including the well-being index, gender, level of education, self-rated health, presence of depression, pain-related limitations, need for home care, need for hospital treatment, visits to specialist doctors, unmet healthcare needs due to financial reasons, age, mental health score, and social support score, a binary logistic regression model was employed. The variables that retained statistical significance in this model are presented in Table 3. Participants who completed high school had 56.5% lower odds of experiencing difficulties in concentration or memory compared to those without a completed elementary education which indicated a negative association between educational level and cognitive problems. Similarly, patients who visited a specialist doctor within the last 12 months had 72.8% higher odds of reporting difficulties with memory and concentration compared to those who visited a specialist more than 12 months ago. This indicated a positive association between the timing of specialist doctor visits and cognitive problems.

**Table 3:** Cross-relationship of odds and 95% confidence intervals of predictors for difficulties with memory or concentration.

Variables	Univariate model		Multivariate model	
	OR (95%CI)	*p* ^*^	OR (95%CI)	p*
**Age**	1.105(1.093–1.117)	<0.001	1.069(1.045–1.094)	<0.001
**Education level**				
No formal education	1		1	
Incomplete primary school	0.394(0.283–0.547)	<0.001	0.709(0.387–1.299)	0.266
Primary school	0.248(0.181–0.339)	<0.001	0.827(0.454–1.507)	0.535
Secondary school	0.135(0.099–0.184)	<0.001	0.435(0.229–0.827)	<0.05
Higher or vocational school	0.116(0.081–0.166)	<0.001	0.577(0.280–1.189)	0.136
Master’s or doctorate	0.023(0.005–0.097)	<0.001	0.149(0.024–0.911)	<0.05
**When was the last time you visited a specialist doctor?**				
Less than 12 months ago	1		1	
More than 12 mounts ago	1.295(1.119–1.499)	<0.005	1.728(1.223–2.441)	<0.05
Never	0.559(0.375–0.833)	<0.005	0.706(0.290–1.722)	0.445
**Have you experienced any pain in the past four weeks? (very mild pain vs. I have not had any)**				
None	1		1	
Very mild	1.857(1.476–2.335)	<0.001	1.852(1.180–2.906)	<0.05
Mild	2.986(2.391–3.730)	<0.001	1.374(0.872–2.163)	0.170
Moderate	3.783(3.071–4.660)	<0.001	1.578(1.019–2.445)	0.051
Severe	5.235(3.958–6.924)	<0.001	1.204(0.677–2.144)	0.527
Very severe	4.141(2.575–6.662)	<0.001	1.930(0.678–5.424)	0.212
**Mental health score**	1.377(1.338–1.417)	<0.001	1.272(1.202–1.346)	<0.001

1-Reference category.

The confounding analysis revealed significant contribution of age, gender, mental health score, and pain on the associations between healthcare access, education level, and cognitive impairment. Adjusting for these variables led to notable changes in the odds ratios for both visiting a specialist doctor and completing high school. Age, gender, and mental health acted as confounders, contributing the associations in varying degrees. Additionally, pain emerged as a notable confounding variable, particularly contributing the association between education level and cognitive impairment. After adjusting for age, the odds change for both educational level and visiting a specialist doctor in predicting functional limitations regarding memory or concentration was negative. Specifically, the odds ratio decreased after adjustment for age, indicating a more protective effect.This finding suggests that age acted as a negative confounder in the analysis. The results of confounding analysis are presented in Table 4.

**Table 4:** Confounding analysis of healthcare access and education.

Variable adjusted for	When was the last time you visited a specialist doctor?		Education level	
	After adj.	Change	After adj.	Change
Baseline (Before adjustment)	2.019		0.435	
Age	1.864	−0.155	0.220	−0.215
Gender	2.006	−0.013	0.383	−0.052
Mental health score	1.803	−0.216	0.223	−0.212
Have you experienced any pain in the past 4 weeks?	1.391	−0.628	9.187	+8.752

Baseline (Before adjustment): these are the initial values before adjusting for any specific confounding variables; “After Adj.” refers to the odds ratio after adjusting for the confounding variable; “Change” represents the difference between the odds ratios before and after adjustment.

## 4 Discussion

Our study explores variations in age, gender, education, healthcare utilization, and the prevalence of difficulties in memory and concentration among a large number of participants. The findings highlight several important aspects that warrant detailed consideration. We identified that age and gender play a significant role in cognitive functioning. Women are, on average, older than men across all examined categories. The age group of 85–89 years reports a higher percentage of significant difficulties in memory and concentration. This supports the general consensus in gerontological research that cognitive decline typically increases with each year of life, and that the oldest segments of the population are most vulnerable. While the longer lifespan of women partially explains their higher representation in groups with cognitive difficulties, it is important to consider how their circumstances, such as social support and access to healthcare, may contribute to these findings (4). Study from Peru emphasizes the importance of sociodemographic factors, such as education level, income, and engagement in mentally stimulating activities, for the cognitive abilities of older adults (4). The authors indicate that higher levels of education and income can positively contribute cognitive functioning. Similar conclusions are drawn from a 2019 study, which highlights the association between health literacy, sociodemographic factors, and the effectiveness of cognitive training programs in older adults (8). Higher levels of health literacy and favorable sociodemographic conditions, such as education and income, are associated with better outcomes in these programs. Our results indicate that older individuals, as well as those with lower levels of education and income, are more likely to experience cognitive difficulties, which is consistent with the conclusions of previous studies. The study from southern Brazil explores the relationship between cognitive functions, sociodemographic factors, and verbal fluency among active older individuals. Results indicate that lower levels of education and income, as well as older age, correlate with poorer cognitive performance (9). These findings align with our research, which also found that higher frequency of health problems and utilization of specialist care may be indicators of worsening cognitive health.

Our analysis reveals that one-third of the adult population reporting difficulties with memory and concentration suffers from depression. These findings align with similar studies in the literature highlighting a close relationship between depression and cognitive decline (11, 13). Additionally, identifying significant associations between social support, education, healthcare utilization, and cognitive functions, especially in the context of memory and concentration problems in older adults, is consistent with previous research (10, 13). Sloane introduces the term “slowing” or “reduced processing speed” in the context of cognition in older adults, often observed as aging progresses. This phenomenon involves slower information processing in the brain, affecting perception, attention, memory, and thinking (9). Factors such as health problems, depression, lack of physical activity, and the presence of multimorbidity can contribute to reduced processing speed in older adults.

Subjective perception of health plays a crucial role in self-assessment of an individual’s health status. Participants who reported the greatest difficulties in cognitive aspects often rated their health as poor or even very poor. This may be due to the interaction between cognitive difficulties, psychological state, and subjective perception of health. For example, individuals facing significant difficulties in memory and concentration may experience higher levels of stress, anxiety, or depression, which can affect their perception of their own health. Furthermore, subjective perception of health and pain can serve as a form of self-defense or coping mechanism, as evidenced by previous research (16).

The results we presented indicate a significant association between experiencing pain in the last 4 weeks and reporting difficulties with memory or concentration. When considering the perception of memory and concentration difficulties in the context of pain, the question arises of how many of those participants who experienced pain in the past 4 weeks actually suffer from persistent pain. If the pain is temporary, we can assume that the perception of memory and concentration difficulties might also be temporary. However, the severity of pain may also contribute this perception, as our results show that as the severity of pain increases, the percentage of participants reporting difficulties with memory or concentration also increases. This association is supported by the findings of a study from Rico (16), which demonstrated that older adults experiencing pain more frequently report subjective memory problems and exhibit lower cognitive performance. Therefore, while persistent pain is associated with cognitive decline, temporary pain may be linked to temporary cognitive problems.

Our analysis has shown that widows/widowers report a higher level of cognitive decline, up to 72.5%. Social support significantly contributes individuals’ cognitive abilities, especially among older adults. Regular interaction with family, friends, and social groups can provide emotional support, a sense of belonging, and confidence, which positively impacts mental health and cognitive functions. Marriage, as a community of belonging, can have a stimulating effect on the brain and maintain its functionality.

The findings of this research indicate that 95% of participants who reported the greatest difficulties also confirmed the presence of health problems in the last 6 months. Correlation was observed between recent visits to specialists and a 72.8% higher risk of memory/concentration problems. However, this connection may not imply a direct causal relationship between healthcare utilization and cognitive decline but rather a higher prevalence of health issues among those seeking specialist care. Epidemiological studies support this notion, showing a link between chronic conditions such as heart failure and atrial fibrillation and cognitive decline in older adults (23, 24).

Results presented suggest the need for non-pharmacological interventions that may contribute to delaying or improving cognitive functions in older adults. The Finnish Intervention Study to Prevent Cognitive Impairment and Disability (FINGER) (24) highlighted success in slowing cognitive decline through an approach involving Mediterranean diet, exercise, social interaction, computer games, and treatment of vascular risk factors. Promoting healthy lifestyle habits, including proper nutrition, regular physical activity, and mental stimulation, represents a key strategy in combating cognitive decline during the aging process. Activities that promote cognitive reserve, such as solving complex puzzles or learning new skills, emerge as vital factors in preserving and protecting mental abilities in older adults. Self-reported difficulties with memory and concentration can be considered important symptoms of cognitive impairment, potentially significantly impacting individuals’ well-being and functioning. Social interaction and active participation in social activities have a significant impact on cognitive well-being, emphasizing the importance of social support in the aging process and maintaining mental health. At the same time, early identification and adequate intervention in the domain of mental health are essential for recognizing and effectively managing initial signs of cognitive decline, highlighting the need for a systematic approach to prevention and treatment of these conditions. Further scientific research, focusing on mechanisms of preserving cognitive functions and developing innovative prevention and intervention strategies, provide the foundation for enhancing understanding and practice in the field of gerontological neurology and public health.

### 4.1 Limitations

Our study is based on the 2019 National Health Survey data, however, like any research, this study also has its limitations that may affect the interpretation and generalization of the results. Since the study is cross-sectional, it can identify associations between sociodemographic and health aspects and cognitive abilities, but cannot establish causative relationships. Longitudinal studies would be more appropriate for understanding the dynamics of changes in cognitive abilities over time. Data on cognitive abilities were collected through participant self-assessments, which may lead to subjectivity, as well as to the fact that questionnaires were filled out by family members or caregivers, taking into account other limitations and disabilities in this population. More objective measures, such as standardized cognitive tests, provide more accurate information about cognitive status. Although the study is based on a national survey, specific groups, such as individuals living in isolated rural areas or those who are institutionalized, were not sufficiently represented. The study may not have included all relevant sociodemographic and health factors that may affect cognitive abilities. For example, genetic factors, family medical history, and more detailed aspects of lifestyle (such as diet and physical activity) may also have a significant impact. Since the data were collected in 2019, it is possible that relevant sociodemographic and health factors have changed, especially considering the impact of the COVID-19 pandemic on the health and well-being of the population. The study may not have fully taken into account the contribution of cultural and economic factors specific to Serbia, which can have a particular impact on cognitive abilities and access to healthcare. Understanding these limitations is crucial for interpreting the study results and for planning future research that could contribute to a deeper understanding of the factors influencing the cognitive abilities of the older adult population in Serbia.

## 5 Conclusion

Given the increasingly aging global population, understanding the factors contributing to the preservation of cognitive abilities becomes increasingly important. Our study, based on the 2019 national health survey of Serbian residents, provides significant insights into the complex interaction among sociodemographic characteristics, health status, and cognitive abilities among older adults. The results indicate that factors such as education, social support, and access to healthcare significantly contribute cognitive abilities, emphasizing the need for a comprehensive approach in promoting cognitive health. Higher levels of education may be associated with better cognitive abilities, while social support and regular contacts with the healthcare system have been identified as key factors in preserving cognitive functions. However, the study also acknowledges limitations, including its cross-sectional nature, which precludes establishing causal relationships, as well as potential subjective biases in self-assessment of cognitive abilities. Future research should incorporate longitudinal studies to better understand the dynamics of cognitive changes, as well as more objective measures of cognitive abilities. We believe that a multidisciplinary approach is necessary in promoting cognitive health. Through the integration of educational, social, and healthcare strategies, we can work toward improving the quality of life for older adults, promoting healthy aging, and preserving cognitive functions.

## Data availability statement

The raw data supporting the conclusions of this article will be made available by the authors, without undue reservation.

## Ethics statement

Ethical review and approval was not required for the study on human participants in accordance with the local legislation and institutional requirements. Written informed consent from the patients/participants or the patients’/participants’ legal guardian/next of kin was not required to participate in this study in accordance with the national legislation and the institutional requirements.

## Author contributions

AM: Writing – original draft, Writing – review & editing. SvR: Supervision, Validation, Visualization, Writing – review & editing, Resources. SnR: Supervision, Validation, Writing – review & editing. IS: Supervision, Validation, Visualization, Writing – review & editing. KJ: Supervision, Visualization, Writing – review & editing. SI: Supervision, Validation, Writing – review & editing. OD: Validation, Visualization, Writing – review & editing. GD: Supervision, Validation, Writing – review & editing. JR: Validation, Writing – review & editing. VS: Validation, Supervision, Writing – review & editing. NS: Supervision, Validation, Writing – review & editing. AG: Formal analysis, Validation, Software, Writing – review & editing.

## References

[1] World Health Organisation (2012). 10 facts on ageing and the life course. Available at: https://www.who.int/news-room/fact-sheets/detail/10-facts-on-ageing-and-health (Accessed January 26, 2023).

[2] Statistical Office of the Republic of Serbia. (2022). Census of Population, Households and Dwellings in the Republic of Serbia 2021: Results by Municipalities and Cities. [online] Available at: https://data.stat.gov.rs/Home/Result/3104021001?languageCode=sr-Latn (Accessed 10 December 2023).

[3] World Economics. (n.d.). Serbia: median age. Available at: https://www.worldeconomics.com/Demographics/Median-Age/Serbia.aspx

[4] Chino B Zegarra-Valdivia J de Frutos-Lucas J Paredes-Manrique C Custodio N. Impact of sociodemographic features and lifestyle on cognitive performance of Peruvian adults. J Alzheimers Dis. (2022) 90:599–608. doi: 10.3233/JAD-220428, PMID: 36155510 PMC9697037

[5] United Nations. (1946). Constitution of the World Health Organization. Available at: https://apps.who.int/gb/bd/PDF/bd47/EN/constitution-en.pdf

[6] WHO. Mental health: strengthening mental health promotion, (2001). Fact Sheet No. 220 Geneva, Switzerland, 2001. Updated August 2014. Available at: http://www.who.int/mediacentre/factsheets/fs220/en/

[7] Sloane PD Warshaw G. Should slowing be considered a distinct geriatric syndrome? J Am Med Dir Assoc. (2022) 23:20–2. doi: 10.1016/j.jamda.2021.11.028, PMID: 34953590

[8] Verney SP Gibbons LE Dmitrieva NO Kueider AM Williams MW Meyer OL. Health literacy, sociodemographic factors, and cognitive training in the active study of older adults. Int J Geriatr Psychiatry. (2019) 34:563–70. doi: 10.1002/gps.5051, PMID: 30548889 PMC6557659

[9] Souza MC Bernardes FR Machado CK Favoretto NC Carleto NG do E SC. Relationship between cognitive and sociodemographic aspects and verbal fluency of active elderly. Rev CEFAC. (2018) 20:493–502. Available from: doi: 10.1590/1982-0216201820417717

[10] Bernardelli G Caruso P Travaini G Merzagora I Gualdi F Sartori RDG. Socio-demographic characteristics and cognitive performance in oldest old subjects asking for driving license renewal. BMC Geriatr. (2020) 20:241. doi: 10.1186/s12877-020-01637-1, PMID: 32652945 PMC7353803

[11] Tetsuka S. Depression and dementia in older adults: a neuropsychological review. Aging Dis. (2021) 12:1920–34. doi: 10.14336/AD.2021.0526, PMID: 34881077 PMC8612610

[12] Dias NS Barbosa IG Kuang W Teixeira AL. Depressive disorders in the elderly and dementia: an update. Dement Neuropsychol. (2020) 14:1–6. doi: 10.1590/1980-57642020dn14-010001, PMID: 32206191 PMC7077867

[13] Wiels W Baeken C Engelborghs S. Depressive symptoms in the elderly-an early symptom of dementia? A Systematic Review. Front Pharmacol. (2020) 11:34. doi: 10.3389/fphar.2020.00034, PMID: 32116710 PMC7020568

[14] Ownby RL Crocco E Acevedo A John V Loewenstein D. Depression and risk for Alzheimer disease: systematic review, Meta-analysis, and Metaregression analysis. Arch Gen Psychiatry. (2006) 63:530–8. doi: 10.1001/archpsyc.63.5.530, PMID: 16651510 PMC3530614

[15] Domenichiello AF Ramsden CE. The silent epidemic of chronic pain in older adults. Prog Neuro-Psychopharmacol Biol Psychiatry. (2019) 93:284–90. doi: 10.1016/j.pnpbp.2019.04.006, PMID: 31004724 PMC6538291

[16] Bell TR Pope CN Downer B Barba C Crowe M. Pain associates with subjective memory problems and cognition in older Puerto Rican adults. Neuropsychol Dev Cogn B Aging Neuropsychol Cogn. (2022) 29:985–99. doi: 10.1080/13825585.2021.1947957, PMID: 34187312 PMC8716642

[17] Gunnarsson H Agerström J. Pain and social cognition: does pain lead to more stereotyped judgments based on ethnicity and age? Scand J Pain. (2020) 20:611–21. doi: 10.1515/sjpain-2019-0141, PMID: 32101530

[18] MerchantRAAuLSeetharamanSNgSENathaniaJLimJY. Association of Pain and Impact of dual-task exercise on function, cognition and quality of life. J Nutr Health Aging. (2021) 25:1053–63. doi: 10.1007/s12603-021-1671-x, PMID: 34725661

[19] European Health Interview Survey (EHIS wave 3), Methodological Manual, Eurostat, (2018). Available at: https://ec.europa.eu/eurostat/web/products-manuals-and-guidelines/-/KS-02-18-240. (Accessed September 17, 2023).

[20] Kocalevent RD Berg L Beutel ME Hinz A Zenger M Härter M. Social support in the general population: standardization of the Oslo social support scale (OSSS-3). BMC Psychol. (2018) 6:31. doi: 10.1186/s40359-018-0249-9 30016997 PMC6050647

[21] Rutstein SO Johnson K. The DHSWealth index. In: DHS comparative reports no. 6. Calverton, MD, USA: ORC macro. (2004). Available at: http://dhsprogram.com/pubs/pdf/CR6/CR6.pdf

[22] Eurostat, EU-SILC and ECHP Surveys. Distribution of income by quantiles; Eurostat: Brussels, Belgium, (2016). Available at: https://ec.europa.eu/eurostat/en/web/products-datasets/-/ILC

[23] Morley JE. Cognition and chronic disease. J Am Med Dir Assoc. (2017) 18:369–71. doi: 10.1016/j.jamda.2017.02.010 28433119

[24] Ngandu T Lehtisalo J Solomon A Levälahti E Ahtiluoto S Antikainen R. A 2 year multidomain intervention of diet, exercise, cognitive training, and vascular risk monitoring versus control to prevent cognitive decline in at-risk elderly people (FINGER): a randomised controlled trial. Lancet. (2015) 385:2255–63. doi: 10.1016/S0140-6736(15)60461-5 25771249

